# Durability of Abstinence After Completing a Comprehensive Digital Smoking Cessation Program Incorporating a Mobile App, Breath Sensor, and Coaching: Cohort Study

**DOI:** 10.2196/25578

**Published:** 2021-02-15

**Authors:** Jennifer D Marler, Craig A Fujii, Joseph A Galanko, Daniel J Balbierz, David S Utley

**Affiliations:** 1 Carrot Inc. Redwood City, CA United States; 2 Biostatistics Core for the Center for Gastrointestinal Biology and Disease and the Clinical Nutrition Research Center Department of Medicine, Division of Gastroenterology and Hepatology University of North Carolina at Chapel Hill Chapel Hill, NC United States

**Keywords:** smoking cessation, digital health, smartphone, digital sensor, carbon monoxide, breath sensor, biofeedback, mobile apps, health promotion, app

## Abstract

**Background:**

Despite decreasing prevalence over the last several decades, cigarette smoking remains the leading cause of preventable death and disease, underscoring the need for innovative, effective solutions. Pivot is a novel, inclusive smoking cessation program designed for smokers along the entire spectrum of readiness to quit. Pivot leverages proven methods and technological advancements, including a personal portable breath carbon monoxide sensor, smartphone app, and in-app text-based coaching. We previously reported outcomes from the end of active Pivot program participation in 319 adult smokers. Herein, we report longer-term follow up in this cohort.

**Objective:**

The aim of this study was to assess and report participant outcomes 3 months after completion of Pivot, including smoking behavior, quit rates, continuous abstinence rates and durability, and predictors of abstinence.

**Methods:**

This prospective remote cohort study included US-based cigarette smokers aged 18 to 65 years who smoked ≥5 cigarettes per day (CPD). Three months after completion of active participation in Pivot, final follow-up data were collected via an online questionnaire. Outcomes included smoking behavior (CPD and quit attempts), self-reported quit rates (7- and 30-day point prevalence abstinence [PPA]), and continuous abstinence rates (proportion who achieved uninterrupted abstinence) and duration. Exploratory regression analyses were performed to identify baseline characteristics associated with achievement of 7-day PPA, 30-day PPA, and continuous abstinence.

**Results:**

A total of 319 participants completed onboarding (intention-to-treat [ITT]); 288/319 participants (90.3%) completed follow up (completers) at a mean of 7.2 (SD 1.2) months after onboarding. At final follow up, CPD were reduced by 52.6% (SE 2.1; *P*<.001) among all 319 participants, and most completers (152/288, 52.8%) reduced their CPD by at least 50%. Overall, most completers (232/288, 80.6%) made at least one quit attempt. Quit rates increased after the end of Pivot; using ITT analyses, 35.4% (113/319) achieved 7-day PPA and 31.3% (100/319) achieved 30-day PPA at final follow up compared with 32.0% (102/319) and 27.6% (88/319), respectively, at the end of the Pivot program. Continuous abstinence was achieved in about a quarter of those who onboarded (76/319, 23.8%) and in most who reported 30-day PPA at the end of Pivot (76/88, 86.4%), with a mean abstinence duration of 5.8 (SD 0.6) months. In exploratory regression analyses, lower baseline CPD, more positive baseline attitudes reflecting higher self-efficacy (higher confidence to quit and lower perceived difficulty of quitting), and higher education were associated with achieving abstinence.

**Conclusions:**

This study provides the first longer-term outcomes of the Pivot smoking cessation program. At final follow up, quit rates increased and continuous abstinence was favorable; the majority who achieved abstinence at the end of Pivot sustained abstinence throughout follow up. Decreases in CPD persisted and most participants made a quit attempt. Overall, final follow-up outcomes were stable or improved when compared to previous outcomes from the end of the program. These findings validate earlier results, and suggest that Pivot is an effective and durable solution for smoking cessation.

**Trial Registration:**

ClinicalTrials.gov NCT03295643; https://clinicaltrials.gov/ct2/show/NCT03295643

## Introduction

### Background

Tobacco use, primarily through cigarette smoking, is the leading cause of preventable disease, disability, and mortality in the United States [1]. Although smoking has declined over the last several decades, it remains a significant public health problem; in 2019, 14.0% of US adults (34.1 million people) were still current cigarette smokers [2].

Quitting smoking is one of the most important steps one can take for their health and can add as much as a decade to life expectancy; accordingly, most smokers (approximately 70%) want to quit [3,4]. Proven smoking cessation treatments include behavioral counseling and pharmacotherapy, which are now widely available. Use of these evidence-based approaches increases the rate of quitting by at least 40% [5-8]. However, use remains low, with less than one-third of smokers using any proven cessation treatments (eg, behavioral counseling, medication). As a result, most quit attempts are unassisted and more than 90% of these attempts are unsuccessful [4].

With room to expand and improve treatment options, the last decade has seen novel approaches to smoking cessation, including mobile and web-based options. A 2019 meta-analysis by Whittaker et al [9] assessed phone text messaging and app-based interventions for smoking cessation. In an assessment comprising 13 studies with 14,133 participants, the authors reported that automated text messaging interventions were more effective than minimal smoking cessation support (relative risk [RR] 1.54, 95% CI 1.19-2.00; I^2^=71%). The authors also assessed five studies comprising 3079 participants, comparing a smoking cessation smartphone app with lower-intensity smoking cessation support (either a lower-intensity app or nonapp minimal support). This assessment provided no evidence that smartphone apps improved the likelihood of smoking cessation (RR 1.00, 95% CI 0.66-1.52; I^2^ = 59%), but the authors noted that the evaluated evidence was of very low certainty due to inconsistency and imprecision, highlighting the need for more randomized controlled trials (RCTs) in this area. More recently, Bricker et al [10] performed an RCT comparing an acceptance and commitment therapy–based smoking cessation smartphone app (iCanQuit, n=1214) with a United States Clinical Practice Guideline (USCPG)-based app (QuitGuide, n=1201). At 12 months after randomization, iCanQuit participants had 1.49 times higher odds of quitting smoking compared with that of QuitGuide participants (28.2%, 293/1040 vs 21.1%, 225/1067; odds ratio [OR] 1.49, 95% CI 1.22-1.83; *P*<.001).

The Pivot program is a novel digital health intervention for smoking cessation that seeks to expand on these previous findings with respect to both intervention design and associated outcomes. Pivot comprises a multiphase mobile app, as well as the first Food and Drug Administration (FDA)-cleared personal carbon monoxide (CO) breath sensor, and dedicated human coaching delivered through in-app text messaging. Pivot is designed for individuals with varying levels of readiness to quit, and is based on the USCPG for treating tobacco use and dependence.

A prospective cohort study evaluated outcomes in 319 adult smokers who underwent the Pivot program (intention to treat [ITT] cohort); 272 (85.3%) participants completed the end-of-Pivot questionnaire (completer cohort) [11]. The study included individuals along the spectrum of readiness to quit; at study entry, the majority of participants (66.5%, 212/319) were not planning on quitting smoking in the next 30 days. Participant engagement, changes in attitudes toward quitting smoking, and changes in smoking behavior during and at the end of the Pivot program (mean 4.1, SD 1.4 months after enrollment) were assessed. Participants had a mean of 12.4 (SD 7.1) weeks of active program engagement, defined as at least one of the following per week: completing a breath sample; logging a cigarette; starting or completing a daily activity, challenge, or check-in; or messaging one’s coach. Repeated-measures linear mixed-model analyses demonstrated positive changes in attitudes at the end of the prequit portion of the program, with increased confidence to quit (4.2 to 7.4, *P*<.001) and decreased expected difficulty in maintaining quit (3.1 to 6.8, *P*<.001). The quit attempt rate (ie, those making ≥1 quit attempt lasting ≥1 day) was 79.4% (216/272, completer analysis). At the end of Pivot, 7-day point prevalence abstinence (PPA) rates were 32.0% (102/319, ITT analysis) and 37.5% (102/272, completer analysis); 30-day PPA rates were 27.6% (88/319, ITT) and 32.4% (88/272, completer). Moreover, 30-day PPA rates were comparable among those ready and not ready to quit in the next 30 days at baseline. Of those not achieving abstinence, 25.9% (44/170, completer) achieved ≥50% reduction in cigarettes per day (CPD) at the end of the Pivot program.

Although these data are encouraging, there is an ongoing need to assess the durability of short-term results, and thereby establish longer-term outcomes in novel smoking cessation programs such as Pivot.

### Objectives

This report focuses on participant outcomes 3 months after the completion of Pivot, including smoking behavior, quit rates, continuous abstinence rates and durability, and predictors of abstinence.

## Methods

### Study Design

This was a prospective, open-label single-arm cohort study performed with institutional review board (IRB) approval. The study was performed remotely on an ambulatory basis. Study participants participated in the Pivot program and completed online study questionnaires. A detailed description of the study methodology was previously provided, with initial focus on outcomes at the end of active participation in Pivot [11].

### Consent and Ethical Approval

All participants provided electronic informed consent before participation. The study was reviewed and approved by Solutions IRB (protocol number 2017/09/22) and was registered with Clinicaltrials.gov (NCT03295643).

### Pivot Program

Pivot is a self-paced, comprehensive digital smoking cessation solution that includes an over-the-counter CO breath sensor, the multiphase Pivot mobile app, and human coaching delivered one-on-one through in-app text messaging [11].

Pivot Breath Sensor is a personal interactive FDA-cleared device that measures CO in exhaled breath. In line with wearable devices, the CO breath sensor provides real-time personal biometric data to users, enabling them to link their smoking behavior and CO values and track their progress in reducing or quitting smoking. This leverages the findings of several published studies [12-15] as well as expert opinion [16,17], which suggest that personal CO breath sample data can be educational and motivational, and may lead to changes in attitudes toward quitting and smoking behavior. To that end, the CO breath sensor is incorporated in the Pivot program as an engagement tool, with the intention that users will find their expired CO values informative and motivational.

In the multiphase Pivot app, participants could log cigarettes, follow trends in their CO values, complete educational and preparatory activities, set a quit date, make a quit plan, undertake short-term practice quits, learn about FDA-approved cessation medications, complete daily check-ins upon quitting smoking, and communicate with their coach.

Coaching was undertaken through asynchronous in-app text messaging, thus allowing participants to respond to coach-initiated contact or to initiate contact with their coach whenever it was convenient for them. Pivot coaches are trained specialists in tobacco cessation. The coach and Pivot participant are paired for the duration the participant is in Pivot to foster rapport and continuity. Coach-initiated contact included outreach 3 times a week from entry through the first 30 days after the quit date, once per week for the next 30 days, and then every other week for the last 30 days. Participants could initiate contact with their coach as frequently as desired.

The Pivot program’s foundation is evidence-based and applies the USCPG-recommended “5 As” (Ask, Advise, Assess, Assist, and Arrange); tailors to one’s readiness to quit smoking [18]; encourages the use of FDA-approved pharmacotherapy [18-21]; uses effective methods and supportive theories for smoking cessation (eg, motivational interviewing, cognitive behavioral therapy, and self-determination theory) [18,22-24]; and provides behavioral counseling through a live, dedicated coach [6,18,21,25].

### Eligibility

To be eligible for participation, individuals had to meet all of the following eligibility criteria: 18-65 years of age, English-speaking, smoke ≥5 CPD, own and use a smartphone that is compatible with the Pivot app and breath sensor software (iPhone 5 and above, operating system iOS 9.0 and above, or Android operating system 4.4 and above), be employed for ≥20 hours a week, and live in the United States. Although we aim for broad availability of Pivot through multiple channels such as private and public insurers, direct-to-consumer, and not-for-profit foundations, at the time this study was performed, Pivot was initially only available to individuals through their employers (self-insured employers or employee wellness programs). As such, the employment requirement was applied to assess Pivot in individuals closely aligned with Pivot’s initial user population. 

### Study Procedure

Study participants completed an online screening form, a screening phone call, electronic informed consent, web registration, and the baseline electronic questionnaire. They were mailed the breath sensor, which they set up independently using the labeling. Technical support was available as needed. Participants were assigned a coach with whom they worked for the duration of their participation in Pivot. Over the entire study, participants were compensated US $10 to $50 per completed study questionnaire and US $50 for returning the CO breath sensor for up to a total of US $315, using Visa gift cards. Specifically, for this follow-up portion of the study, participants were compensated US $50 for completing the final study questionnaire and US $50 for returning Pivot Breath Sensor if they had not yet done so. Compensation was not associated with use of the various components of Pivot, level of engagement, or smoking/quitting status.

### Data Collection

Data were collected electronically through participant input in the Pivot online registration form, Pivot app, and online questionnaires. Study data were imported directly into a secure database (PostgreSQL, PostgreSQL Global Development Group).

### Outcomes

Outcomes from this follow-up phase of the study focus on smoking behavior, quit rates, continuous abstinence rates and duration, and predictors of abstinence. For smoking behavior, outcomes include CPD and quit attempts. A quit attempt was defined as going at least 1 day without smoking cigarettes, even a single puff. Quit rates were self-reported and include 7- and 30-day PPA. Participants were considered to have achieved 7-day (30-day) PPA if they answered “no” to the following question: “In the last 7 (30) days have you smoked any cigarettes, even a single puff?” As the Pivot program has no face-to-face contact, and data collection is achieved through remote means using the app and electronic questionnaires, biochemical verification of smoking status was not pursued in accordance with previous recommendations [26].

Although not listed as a preregistered outcome on Clinicaltrials.gov, we also evaluated continuous abstinence. The rationale for including continuous abstinence was to enhance the ability to compare outcomes in this study to those in other studies, to include an outcome that was a closer proxy for lifelong abstinence than PPA, and to include an outcome that was temporally closer to the Pivot program intervention than PPA [27]. Continuous abstinence includes the proportion of participants who achieved uninterrupted abstinence; a conservative definition was applied in which there was no grace period after the onset of abstinence, and any smoking (even a single puff) precluded designation of continuous abstinence. To be considered continuously abstinent, one had to report 30-day PPA on the end-of-Pivot questionnaire, 30-day PPA on the final questionnaire, and indicate a duration of abstinence that was equal to or greater than the number of days between the two questionnaires plus an additional 30 days. The average duration of abstinence is reported in those who achieved continuous abstinence. Finally, exploratory regression analyses were performed to identify the baseline characteristics associated with achievement of 7-day PPA, 30-day PPA, and continuous abstinence on the final questionnaire.

All participants were sent the end-of-Pivot and final follow-up questionnaires, regardless of their progress or completion status in the Pivot program.

### Sample Size

Sample size was previously addressed [11]. A previous evaluation showed that attitudes toward quitting (motivation to quit, confidence to quit) are meaningful predictors of quit attempts [28]. On the basis of a previous assessment of 41 individuals using the first stage of Pivot (Explore), we estimated that the mean change in ratings assessing attitudes toward quitting (confidence to quit and expected difficulty maintaining quit) would be ≥1 (SD 4) just before reaching the Quit stage of the program [29]. On the basis of these estimates, there was 80% power to detect a significant difference in these ratings with a sample size of 101. As this was an initial study of the complete Pivot program, and in the context of known high attrition rates with mobile health apps [30-32], we applied conservative retention estimates drawn from other similar studies. Specifically, the target enrollment of 310 was estimated to yield at least 100 participants still engaged at the end of the Pivot program. The study enrolled 319 participants (ITT cohort) and 288 participants completed the final follow-up questionnaire (completer cohort).

### Analyses

Changes in CPD were assessed from baseline to the final follow-up questionnaire. Participants served as their own controls, and comparisons were made to no change. To evaluate changes in CPD, repeated-measures linear mixed-model analyses were performed using a compound symmetric correlation matrix to model the repeated measures within participants. Because these measurements were taken at the same point in the study (not necessarily after the same amount of time, as progression through Pivot is self-paced), study stage (baseline vs final follow up) was used as a surrogate for time. To make specific comparisons across time, *F* statistics were computed using the results from the model.

Analyses were conducted to calculate the mean (SD) for normally distributed variables for actual data or mean (SE) for modeled data. Median (IQR) values were used in instances of non-normally distributed variables. A paired one-sample *t* test was used for numeric data. The Fisher exact or χ^2^ test was used for comparisons of categorical data. The McNemar test was used for two-category match-paired data. Cohen κ statistic was used for three-category match-paired data.

In the assessment of quit rates (PPA), two sets of analyses were performed. In the ITT analysis, individuals who did not respond to PPA questions were assumed to be smoking. A study completer analysis was also performed, which only included individuals who completed the final follow-up questionnaire. Participants were sent the final follow-up questionnaire regardless of whether or not they completed the Pivot program. For additional assessments performed at the end of the study (quit attempts, proportion who reduced CPD by at least 50%), a study completer analysis was performed. This analysis approach comports with previous reports assessing app-based digital cessation programs [33,34].

We performed exploratory post-hoc analyses using univariate logistic regression to explore associations between baseline characteristics and smoking behavior outcomes. We evaluated each independent baseline variable as a predictor in a separate model, with the binary outcomes of 7-day PPA, 30-day PPA, and continuous abstinence. We then performed multivariate logistic regression using forward selection of baseline variables with the binary outcomes of 7-day PPA, 30-day PPA, and continuous abstinence. Analyses were conducted using SAS Version 9.4 (SAS Institute, Cary, NC). Statistical significance was set at *P*<.05.

## Results

### Enrollment and Questionnaire Completion

**Figure 1:**
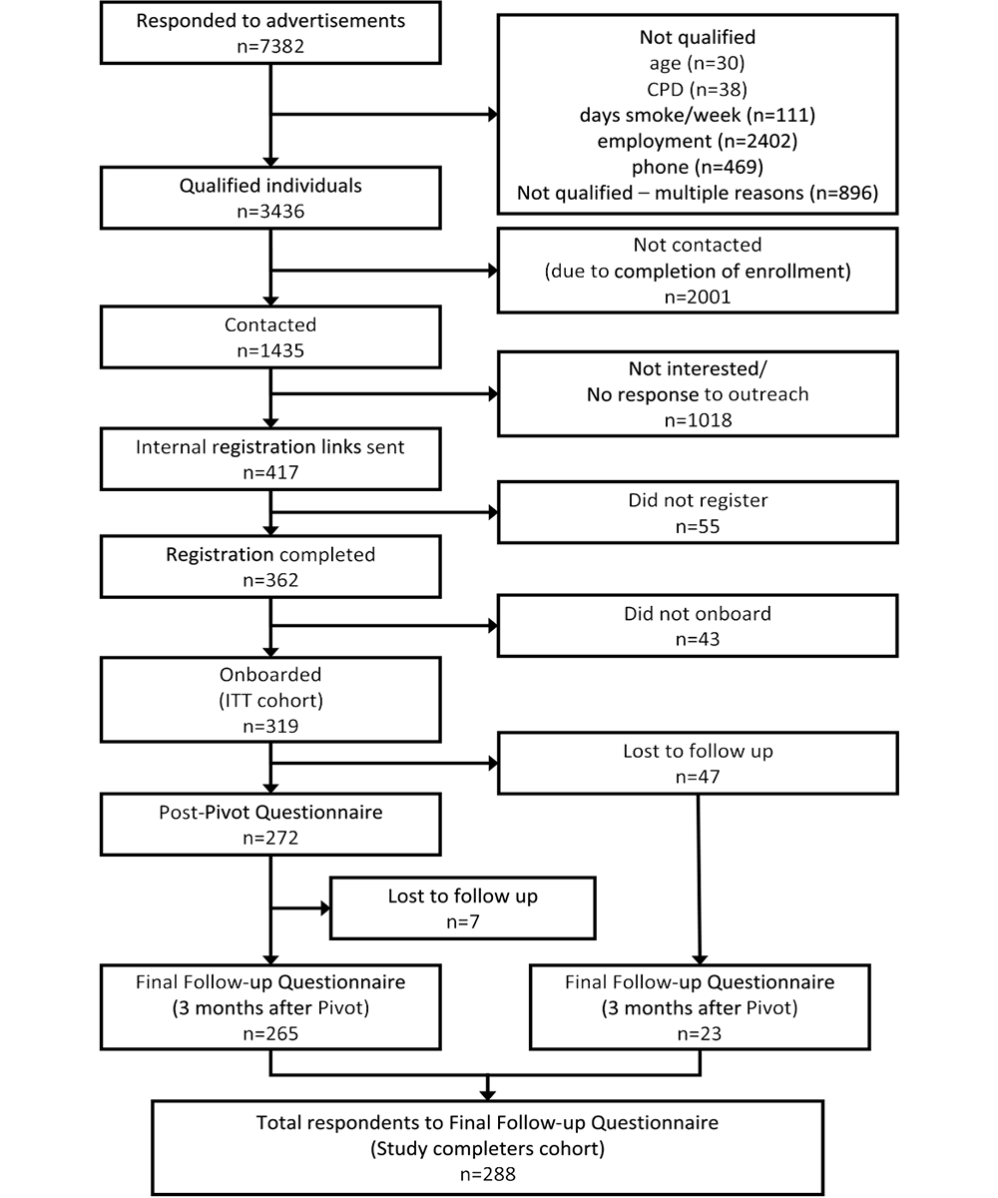
Study participant flow: Consolidated Standards of Reporting Trials (CONSORT) diagram. ITT: intention to treat; CPD: cigarettes per day.

A total of 319 participants completed onboarding and comprised the ITT cohort. At the end of active participation in Pivot, at a mean of 4.1 (SD 1.4) months after enrollment, 85.3% (272/319) of the participants completed the post-Pivot questionnaire [9]. At the end of the final follow-up period (3 months after completion of Pivot), at a mean of 7.2 (SD 1.2) months after enrollment, 90.3% (288/319) of participants completed the final follow-up questionnaire, who comprise the study completers cohort in this report. Study enrollment and attrition are depicted in the participant flow diagram shown in Figure 1.

### Baseline Characteristics

The study sample comprised 57.7% (184/319) women, had a mean age of 42.8 (SD 10.2) years, smoked a mean of 17.7 (SD 7.6) CPD at baseline, and had been smoking for a mean of 26.4 (SD 10.7) years. Participants represented 47 of the 50 US states; North Dakota, Nevada, and Arkansas were not represented. At baseline, 33.5% (107/319) of participants indicated that they were seriously thinking of quitting smoking in the next 30 days, 63.0% (201/319) indicated they were seriously thinking of quitting in the next 6 months, and 3.5% (11/319) indicated they were not thinking of quitting smoking. On average, participants had made 2.1 (SD 3.3) quit attempts over the past 12 months.

### Smoking Behavior

Repeated-measures linear mixed-model analysis was performed with estimated final follow-up CPD values compared with baseline. There was an estimated 52.6% (SE 2.1) reduction in CPD at final follow up (*P*<.001), which persisted from the end of Pivot. Table 1 details the CPD at baseline, the end of Pivot, and at final follow up.

Among those who completed the final follow-up assessment, most (152/288, 52.8%) reduced their CPD by ≥50%. 

Focusing on the study completers who did not achieve at least 7-day PPA at final follow up (n=175), CPD decreased by 22.7% (SD 37.0), and 22.3% (39/175) reduced their CPD by ≥50%.

As reported previously, among the 170 participants who completed the end-of-Pivot questionnaire and did not achieve abstinence, 25.9% (44/170) achieved ≥50% reduction in CPD. Of these participants, 95.5% (42/44) completed final follow up, at which time 66.7% (28/42) reported 7-day PPA or ≥50% reduction in CPD. Specifically, 26.2% (11/42) achieved 7-day PPA (16.7%, 7/42 also achieved 30-day PPA) and 40.5% (17/42) did not achieve PPA but reported ≥50% reduction in CPD.

Overall, most completers (232/288, 80.6%) reported making at least one quit attempt during the study with an average of 2.9 (SD 3.7) quit attempts made per participant.

**Table 1:** Changes in cigarettes per day (CPD) based on the linear mixed model (N=319).

Time point	CPD		Change in CPD vs baseline		Percent change in CPD vs baseline	
	Mean (SE)	*P* value^a^	Mean (SE)	*P* value^a^	Mean (SE)	*P* value^a^
Baseline	17.7 (0.43)	N/A^b^	N/A	N/A	N/A	N/A
End of Pivot	8.0 (0.45)	<.001	–9.7 (0.42)	<.001	–54.5 (2.2)	<.001
Final follow up	8.5 (0.44)	<.001	–9.2 (0.41)	<.001	–52.6 (2.1)	<.001

^a^Compared to baseline.

^b^N/A: not applicable.

### Quit Rates

Quit rates increased from the end of Pivot to final follow up. Specifically, at final follow up, 35.4% (113/319) achieved 7-day PPA and 31.3% (100/319) achieved 30-day PPA using ITT analysis. These rates increased from those obtained at the end of Pivot, when the 7-day PPA was 32.0% (102/319) and the 30-day PPA was 27.6% (88/319).

Similarly, at final follow up, 39.2% (113/288) achieved 7-day PPA and 34.7% (100/288) achieved 30-day PPA, using the study completer analysis. These rates increased from those obtained at the end of Pivot, when the 7-day PPA was 37.5% (102/272) and the 30-day PPA was 32.4% (88/272).

**Figure 2:**
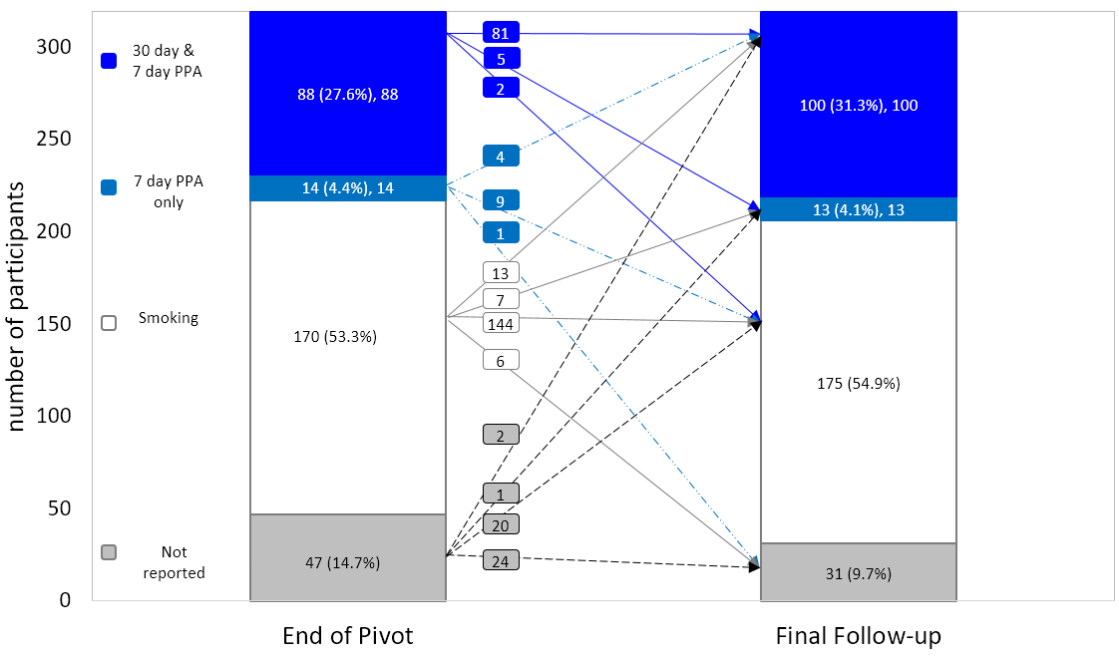
Participant smoking status at the end of Pivot and at final follow up.

Assessing only the 265 participants who completed both the end-of-Pivot and final follow-up questionnaires, 41.5% (110/265) achieved 7-day PPA and 37.0% (98/265) achieved 30-day PPA. Among the 23 participants who did not complete the end-of-Pivot questionnaire but did complete the final follow up, 20 were still smoking, 1 reported 7-day PPA, and 2 reported 30-day PPA (as well as 7-day PPA) at final follow up (Figure 2).

Of the 88 participants who achieved 30-day PPA at the end of Pivot, 92.0% (81/88) reported 30-day PPA at final follow up.

From the end of Pivot to final follow up, there were 23 newly abstinent participants. Specifically, among the 217 participants who had not achieved at least 7-day PPA at the end of Pivot, 10.6% (23/217) reported abstinence at final follow up. Focusing on these 23 individuals, 15 achieved 30-day PPA (as well as 7-day PPA) and 8 achieved 7-day PPA (Figure 2).

Using all available data, 42.0% (134/319, ITT) achieved 7-day PPA and 35.4% (113/319, ITT) achieved 30-day PPA at some point during the study.

### Continuous Abstinence

Continuous abstinence was reported in 76 participants. Table 2 details continuous abstinence rates in various study subgroups.

Among all study participants, approximately a quarter achieved continuous abstinence. Among those who completed the final follow-up questionnaire, just over a quarter achieved continuous abstinence. Focusing on participants who made at least one quit attempt, nearly a third achieved continuous abstinence. Focusing on participants who reported 30-day PPA at the end of Pivot, the continuous abstinence rate was very high. The mean duration of continuous abstinence in this group was 5.8 (SD 0.6) months.

**Table 2:** Continuous abstinence rates in various study subgroups.

Study subgroup	N	Continuous abstinence rate, n (%)
Enrolled (intention to treat)	319	76 (23.8)
Completed final follow-up questionnaire (completers)	288	76 (26.4)
Made at least one quit attempt	232	76 (32.8)
Achieved 30-day point prevalence abstinence at the end of Pivot	88	76 (86.4)

### Predictors of Abstinence

Focusing on all 319 participants who enrolled in the study, exploratory univariate regression analyses were performed to examine associations between participant baseline variables and final follow-up achievement of 7-PPA, 30-day PPA, and continuous abstinence; results are detailed in Table 3.

Lower baseline CPD, higher confidence to quit, and lower perceived difficulty maintaining quit were associated with an increased likelihood of achieving 7-day PPA, 30-day PPA, and continuous abstinence. Non-White Hispanic/Latino/Latina/ Spanish origin was associated with a higher likelihood of achieving 7-day PPA, 30-day PPA, and continuous abstinence; however, this association should be considered with caution due to the low number of participants in this category (15/319, 4.7%).

**Table 3:** Univariate logistic regression analyses of baseline predictors of 7-day PPA, 30-day PPA, and continuous abstinence at final follow up among all study participants (N=319).

Baseline variable		N	7-day PPA^a^		30-day PPA		Continuous abstinence	
			OR^b^ (95% CI)	*P* value^c^	OR (95% CI)	*P* value	OR (95% CI)	*P* value
Age		319	0.99 (0.97-1.01)	.33	0.98 (0.96- 1.00)	.08	0.98 (0.96-1.01)	.12
**Gender**								
	Male	135	1 [Reference]	N/A^d^	1 [Reference]	N/A	1 [Reference]	N/A
	Female	184	1.39 (0.87-2.23)	.17	1.30 (0.80-2.11)	.29	1.08 (0.64-1.83)	.76
**Race/ethnicity**								
	White	264	1 [Reference]	N/A	1 [Reference]	N/A	1 [Reference]	N/A
	American Indian/Alaska Native	4	0.66 (0.07-6.39)	.72	0.78 (0.08-7.62)	.83	1.21 (0.12-11.9)	.81
	Asian	5	0.49 (0.05-4.46)	.53	0.59 (0.06-5.32)	.63	0.91 (0.10-8.28)	>.99
	Black/African American	22	1.36 (0.56-3.31)	.50	1.09 (0.43-2.78)	.85	2.08 (0.83-5.19)	.20
	Hispanic/Latino/Latina/Spanish origin	15	3.93 (1.31-11.9)	.02	3.51(1.21-10.20)	.02	3.18 (1.11-9.13)	.03
	Native Hawaiian or other Pacific Islander	2	1.97 (0.12-31.8)	.63	2.34 (0.15-37.90)	.55	—^e^	—
	Other race, ethnicity, or origin	7	0.79 (0.15-4.14)	.78	0.94 (0.18-4.90)	.94	1.45 (0.28-7.68)	.66
**Education**								
	Bachelor’s degree or greater	96	1 [Reference]	N/A	1 [Reference]	N/A	1 [Reference]	N/A
	Less than bachelor’s degree	223	1.60 (0.95-2.70)	.08	1.44 (0.84-2.45)	.18	1.52 (0.84-2.76)	.16
**Income (US $)**								
	<50,000	136	1 [Reference]	N/A	1 [Reference]	N/A	1 [Reference]	NA
	>50,000	173	0.79 (0.50-1.27)	.33	0.77 (0.47-1.25)	.29	1.21 (0.70-2.10)	.49
	Did not answer	10	1.08 (0.29-4.00)	.91	0.81 (0.20-3.28)	.77	1.73 (0.42-7.14)	.45
Cigarettes per day		319	0.96 (0.93-0.99)	.01	0.97 (0.94-1.00)	.048	0.95 (0.91-0.99)	.01
Years smoking		319	0.99 (0.97-1.01)	.44	0.98 (0.96-1.01)	.18	0.98 (0.95-1.00)	.10
Quit attempts in last 12 months		319	1.03 (0.97-1.11)	.33	1.04 (0.97-1.11)	.32	1.04 (0.97-1.11)	.31
**Stage of change**								
	Yes, within the next 30 days	107	1 [Reference]	N/A	1 [Reference]	N/A	1 [Reference]	NA
	Yes, within the next 6 months	201	0.88 (0.54-1.43)	.59	0.96 (0.58-1.60)	.87	1.15 (0.66-2.00)	.78
	No, not thinking of quitting	11	0.96 (0.26-3.48)	.95	1.23 (0.34-4.48)	.76	0.77 (0.16-3.80)	.35
	Confidence to quit	319	1.11 (1.02-1.21)	.02	1.11 (1.02-1.21)	.02	1.17 (1.06-1.28)	.001
	Perceived difficulty maintaining quit	319	1.14 (1.04-1.25)	.004	1.18 (1.08-1.30)	<.001	1.19 (1.08-1.31)	.003

^a^PPA: point prevalence abstinence.

^b^OR: odds ratio.

^c^*P* values are based on 95% Wald confidence limits.

^d^N/A: not applicable.

^e^—: No participants in this category.

Focusing specifically on the 88 participants who achieved 30-day PPA at the end of Pivot, exploratory univariate regression analyses were performed to examine associations between participant baseline variables and final follow-up achievement of continuous abstinence. None of the evaluated baseline variables was found to be associated with continuous abstinence.

In multivariate regression analyses, lower baseline CPD (OR 0.96, 95% CI 0.93-0.99; *P*=.02), lower perceived difficulty maintaining quit (OR 1.13, 95% CI 1.03-1.24; *P*=.01), and higher education (OR 1.72, 95% CI 1.01-2.94; *P*=.047) were associated with an increased likelihood of achieving 7-day PPA. Lower perceived difficulty maintaining quit (OR 1.18, 95% CI 1.08-1.30; *P*<.001) was associated with achieving 30-day PPA. Lower perceived difficulty maintaining quit (OR 1.13, 95% CI 1.02-1.26; *P*=.02) and higher confidence to quit (OR 1.12, 95% CI 1.01-1.24; *P*=.04) were associated with achieving continuous abstinence. Finally, no baseline variables were predictive of continuous abstinence among the 88 individuals who achieved 30-day PPA at the end of Pivot. 

## Discussion

### Principal Findings

The present report details longer-term follow-up outcomes from a prospective cohort study of 319 adult smokers who underwent the Pivot smoking cessation program. These outcomes, from a mean of 7.2 (SD 1.2) months postenrollment, focus on smoking behavior, quit rates via PPA, continuous abstinence rates and duration, and predictors of abstinence. To our knowledge, this is the first study assessing longer-term outcomes in a mobile smoking cessation program such as Pivot, which includes biofeedback via a personal portable CO breath sensor, education and guidance through a smartphone app, and support through text-based human coaching. At final follow up, CPD were reduced by approximately half, most participants had made a quit attempt, quit rates had increased from the end of the Pivot program, and approximately a quarter of participants achieved continuous abstinence. Regression analyses showed that lower CPD, attitudes toward quitting reflecting higher self-efficacy, and higher education level were associated with achieving abstinence. Overall, final follow-up outcomes persisted or improved when compared to previous outcomes from the end of the Pivot program.

### Specific Findings

#### Scope for Comparison

Comparison with other studies is limited due to the novelty of digital smoking cessation programs that comprise a smartphone app, human-delivered text-based coaching, and a personal biofeedback device. In addition, among the few studies that did include smoking cessation interventions comparable to Pivot, differences in study design or population are significant enough to make comparison challenging. Taking these factors into consideration, we review the outcomes of our study with others below, with consideration of studies that employed a smoking cessation intervention similar to Pivot, expanding the assessment to include a broader group of digital smoking cessation interventions, and finally considering Pivot in the context of different types of smoking cessation interventions.

#### CPD Reduction

At final follow up, participants reduced CPD by 52.6%. Among those who did not achieve PPA, CPD were reduced by 22.7%, and 22.3% (39/175) reduced their CPD by ≥50%. Two additional studies have employed smoking cessation interventions similar to Pivot and included changes in CPD as an outcome. Webb et al [35] performed an RCT in adult smokers in the United Kingdom randomized to a digital therapeutic intervention (treatment, n=265) or very brief advice (control, n=265). The digital therapeutic intervention for smoking cessation comprised a smartphone app delivering cognitive behavioral therapy content, human coaching via phone and in-app chat, craving tools, and tracking capabilities. The control intervention was very brief advice applying the Ask, Advise, Act model. Half of the participants received a personal CO breath sensor that was used to measure their exhaled CO and validate self-reported abstinence. Eligibility criteria included readiness to quit in the next 30 days. Participants had an in-person baseline visit, and then self-reported outcomes via phone or online at 4 weeks after the quit date. Participants set a quit date an average 16 days postrandomization. All participants were offered free nicotine replacement therapy (NRT) for 3 months, which was used by 59.1% (133/225, treatment) and 63.2% (146/231, control) of participants (risk ratio 0.94, 95% CI 0.81-1.08). At 4 weeks post quit date, mean CPD were reduced in those who failed to achieve abstinence by 48.1% in the treatment group and by 48.9% in the control group. The provision of NRT that was used by most participants, the 1-month endpoint that occurred during the active treatment phase of the intervention, and inclusion only of individuals ready to quit smoking in the next 30 days in the Webb study are notable study design differences that likely contribute to differences in CPD reduction from those found in the present study.

The second study that evaluated a smoking cessation intervention similar to Pivot, performed by Krishnan et al [36], was an RCT in which adult smokers in the United States received brief advice along with a personal CO breath sensor and the COach2Quit app (intervention, n=50) or brief advice only (control, n=52). The Coach2Quit app prompted the user to set a quit date, provided reminders to complete two breath samples a day, sent response messages from a text message library to users after breath samples were provided based on their CO results, and provided graphical representation of user CO readings. Eligibility criteria included willingness to set a quit date within 2 weeks of the baseline assessment. Follow-up visits were conducted at 14 days and 30 days from baseline. The median CPD among all participants decreased from 10 at baseline to 5 in the intervention group and to 6 in the control group at 30 days, for an approximate reduction of 40%-50%. This is in range with the 52.6% CPD reduction for all participants found in our study; however, it is unknown if and how CPD reduction changed in the Krishnan study after the active intervention phase.

Looking more broadly at digital health interventions, Garrison et al [37] recently reported results from an RCT comparing the efficacy of 22 days of mobile mindfulness training through the Craving to Quit app with app-based experience sampling (n=143) versus 22 days of app-based experience sampling only (n=182). At 6 months, CPD were reduced but not different between the two groups; the app group reduced CPD by 43.8% (from 16.0, SD 7.1 to 9.0, SD 7.8) and the experience sampling–only group reduced CPD by 45.7% (from 16.2, SD 8.2 to 8.8, SD 9.0). Finally, Alessi et al [38] performed an RCT with 90 participants randomized to usual care and ecological monitoring with abstinence reinforcement (mobile health reinforcement) or without reinforcement (mobile health monitoring). Usual care was 8 weeks of transdermal nicotine and twice-weekly telephone counseling. Ecological monitoring was administered through an interactive voice response system that prompted participants to conduct 1-3 CO breath tests daily, video record the process, and submit the videos. Participants in the abstinence reinforcement group could earn prizes for on-time CO breath tests with CO values consistent with abstinence. At 6 months, CPD were reduced by 47% (baseline 17.6 to 9.3 CPD at 6 months) in the mobile health reinforcement group and by 55% (baseline 20.0 to 9.0 at 6 months) in the mobile health monitoring group.

Overall, assessing the present and aforementioned studies, digital smoking cessation interventions have reported CPD reduction by about half at 6 months. Studies that have assessed interventions similar to Pivot have reported similar reductions achieved earlier, at 1 month; however, the durability of the reduction in these instances is unknown.

#### PPA Rates

PPA between the end of Pivot and final follow up 3 months later increased; using ITT analyses, 35.4% (113/319) achieved 7-day PPA and 31.3% (100/319) achieved 30-day PPA at final follow up, compared to 32.0% (102/319) and 27.6% (88/319), respectively, at the end of the Pivot program.

Comparing our 35.4% final follow-up 7-day PPA rate to other studies with interventions similar to Pivot, Webb et al [35] reported a 44.5% 7-day PPA rate at 1 month. The approximate 35%-45% 7-day PPA range seems within reason when considering that the Webb study included the provision of NRT and required participants to be ready to quit smoking within the next 30 days at enrollment for study eligibility, acknowledging that longer-term data from the Webb study will be informative. Masaki et al [39] performed an RCT of adult smokers recruited from smoking cessation clinics in Japan. Participants were randomized to the intervention, which included a 12-week standard smoking cessation treatment plus the CureApp Smoking Cessation (CASC) system (n=285), or to the control, which consisted of the 12-week standard smoking cessation treatment plus a control app (n=287). The CASC system comprised a smartphone app, paired mobile exhaled CO breath sensor, connected cloud system to upload data, and web-based PC software for physicians. The 12-week standard smoking cessation treatment included five in-person visits with counseling and physician-provided pharmacotherapy of varenicline or nicotine patch. The CASC system and control app were used for 24 weeks. Eligibility criteria included intention to quit smoking immediately. Seven-day PPA at 24 weeks was achieved in 72.3% of the CASC intervention group participants and in 58.2% of the control group participants (*P*<.01). Considering the 58.2% 7-day PPA rate in the control arm, this high 7-day PPA rate in the CASC intervention arm likely reflects what a program similar to Pivot adds when used as a supplement to a traditional intensive smoking cessation program in individuals ready to quit smoking.

Looking more broadly at outcomes from digital smoking cessation interventions, 7-day PPA at 6 months range from 9.8% to 29.6%, with most falling between 17% and 25% [10,37,38,40,41]. Our higher 7-day PPA rate of 35.4% aligns with expectations considering Pivot’s additional features of the personal breath sensor and coaching.

Focusing on 30-day PPA, 31.3% (100/319) achieved this outcome at final follow up in our study. We did not find comparable data in studies that included interventions similar to Pivot. Broadening the scope to include additional digital smoking cessation interventions, 6-month 30-day PPA outcomes ranged from 12.9% to 21.8%, with an additional study reporting a 26.2% 30-day PPA rate at 8 weeks [10,34,40]. Similar to our expectation, we believe that the additional features of Pivot beyond the app likely contributed to the higher 30-day PPA rate.

#### Continuous Abstinence

Continuous abstinence at final follow up was reported in about a quarter of all participants (76/319, 23.8%) and in about a third of those who made a quit attempt (32.8%, 76/232) in our study. Masaki et al [39] reported that 63.9% of participants achieved continuous abstinence at 6 months. These differences in continuous abstinence likely reflect outcomes when Pivot is used as the sole intervention among smokers who represent the entire spectrum of readiness to quit in contrast to when a Pivot-like program is used as an adjunct to an intensive smoking cessation program among individuals ready to quit smoking. Expanding the scope of studies to the broader category of digital smoking cessation interventions, the rates for continuous abstinence at 6 months range from 4.8% to 19.8%, with most falling between 10% and 16% [37,38,40,42]. Overall, there appears to be a trend reflecting the intensity and comprehensiveness of the intervention, with lower-intensity programs reporting continuous abstinence at 6 months in about 10%-16% of participants, mid-intensity programs such as Pivot reporting continuous abstinence in about a quarter of all participants or a third of participants who make a quit attempt, and high-intensity programs achieving continuous abstinence in over half of participants.

Considering Pivot in the context of different types of smoking cessation interventions, we turn to meta-analyses to provide insight on longer-term outcomes. Whittaker et al [9] published a meta-analysis in 2019 assessing automated mobile phone text messaging and app-based interventions for smoking cessation. Long-term abstinence (defined as smoking cessation at 6 months or longer using the most stringent measure available) was higher in text messaging–based interventions compared to minimal smoking cessation support (13 studies, 14,133 participants; RR 1.54, 95% CI 1.19-2.00; I^2^=71%). Evaluating both high- and low-intensity text message–based interventions using data pooled from three studies, the authors reported average ≥6-month abstinence rates of 26.6%-27.1%. A similar effect was not seen for comparison of smartphone app interventions to lower-intensity smoking cessation support (5 studies, 3079 participants; RR 1.00, 95% CI 0.66-1.52; I^2^=59%); however, the authors noted the need for additional data to further assess these interventions, as this particular evaluation comprised 5 studies with many additional studies ongoing at the time of publication.

Matkin et al [43] published a meta-analysis in 2019 on telephone counseling for smoking cessation. In studies that recruited smokers who underwent proactive telephone counseling (ie, counseling that was not delivered through calling a helpline), the counseling increased quit rates (RR 1.25, 95% CI 1.15-1.35; I^2^=52%; 65 trials, 41,233 participants). The authors reported that based on a control group quit rate of 11%, telephone counseling would produce an absolute increase of 2%-4%, resulting in a ≥6-month quit rate of 13%-15%. In a 2017 meta-analysis, Lancaster et al [6] reported that individual counseling increases the likelihood of cessation compared with less intensive support. Based on pooling 27 trials comprising 11,100 participants, individual counseling, when used independently of pharmacotherapy, was estimated to increase cessation by 40% to 80% after at least 6 months. Assuming a control group quit rate of 7% from a brief intervention, individual counseling would be expected to result in an absolute increase of 3%-5%, yielding a 10%-12% quit rate. Finally, in a 2019 meta-analysis, Hartmann-Boyce et al [5] reported that NRT increased quit rates compared to control, by an amount that depended on the baseline quit rate. For example, for an expected quit rate of 3%-5% in people attempting to quit on their own, NRT might increase the quit rate by 2%-3%. However, if the expected quit rate of a population was 15%, another 8% might be expected to quit with NRT use.

Summarizing these findings from meta-analyses, long-term quit rates for various types of smoking cessation interventions include 10%-15% for telephone or individual counseling alone, 26%-27% for automated text messaging, and 5% to more than 23% for NRT alone (depending on the expected quit rate in the population at baseline). Acknowledging that consideration of these data is primarily to establish context for the outcomes of Pivot, and that direct comparison of our cohort study with large meta-analyses is not appropriate, we believe that our 23.8% continuous abstinence rate in all participants is reasonable and encouraging. As a program that includes established evidence-based components such as coaching, as well as novel aspects that aim to leverage nascent but promising approaches to smoking cessation such as biofeedback via a personal breath sensor, we would expect to improve upon existing interventions. Certainly, this should be born out with future additional investigation and data.

#### Abstinence Duration

Hughes et al [44] established the significance of abstinence duration, reporting that abstinence stabilizes at about 6 months. Zhou et al [45] reported similar findings in their evaluation of 2431 smokers who intended to stop smoking in the next 3 months. They followed these individuals periodically for 18 months via internet questionnaires; after 6 months of abstinence, the relapse rate dropped below 20% and the cumulative relapse rate reached a plateau. Herd et al [46] detailed similar findings in a longitudinal survey of 1296 ex-smokers in the general population who quit on their own (not in an interventional study), reporting that a duration of abstinence of 31-182 days was associated with a 58% continuous abstinence rate, whereas duration of abstinence of 183-365 days was associated with a 78% continuous abstinence rate. These data bring further confidence to our results; with a continuous abstinence duration of approximately 6 months (mean 5.8, SD 0.6 months), there is reason to expect stability in our continuous abstinence rate.

Approaching continuous abstinence from a different angle, in a systematic review, Hughes et al [27] assessed the relationship between PPA rates and prolonged abstinence in studies with point prevalence durations of up to 7 days and follow ups of at least 6 months from the quit date. They reported that point prevalence and prolonged abstinence were highly correlated (*r*=0.88) and that prolonged abstinence averaged 0.74 that of PPA, indicating that approximately three-quarters of those who achieve point prevalence will achieve prolonged abstinence. In the most comparable analysis from our data, 79.4% (81/102) of study participants who achieved 7-day PPA at the end of Pivot achieved continuous abstinence of approximately 6 months duration (mean 5.6, SD 0.7 months), findings which align with those reported by Hughes et al [27].

#### Predictors of Abstinence

Exploratory univariate regression modeling demonstrated that among all study participants, lower baseline CPD, higher self-efficacy through confidence to quit, and lower perceived difficulty of maintaining quit were associated with achieving 7-day PPA, 30-day PPA, and continuous abstinence at final follow up, approximately 7 months from enrollment. Multivariate regression modeling found that lower perceived difficulty of maintaining quit was associated with 7- and 30-day PPA and continuous abstinence, lower CPD and higher education were associated with 7-day PPA, and higher confidence to quit was associated with continuous abstinence.

These results are consistent with previous findings. In the aforementioned study of 1296 ex-smokers by Herd et al [46], relapse was associated with lower abstinence self-efficacy. Smit et al [47] assessed predictors of successful quit attempts among 570 smokers motivated to quit in the next 6 months who were randomized to the control group in a web-based smoking cessation intervention study. They reported that self-efficacy was the main factor in predicting quit attempt success.

Nicotine dependence has also been shown to be a predictor of successful cessation [26]. In a systematic literature review of adult general population smokers, Vangeli et al [28] reported that cigarette dependence consistently predicted success after a quit attempt. Hymnowitz et al [48] performed a cohort tracking telephone survey in 13,415 smokers over 5 years. They reported that predictors with the largest RR values for smoking cessation were those associated with nicotine dependence, including CPD. Thereby, it is not surprising that in our study, lower baseline CPD was associated with achieving abstinence.

Finally, higher education has been shown to be a predictor for success in cessation. In a study including 887 smokers undergoing a smoking cessation program through their workplace, a higher educational level (OR 1.81, 95% CI 1.06-3.09, *P*=.03) predicted successful cessation [49]. In a study including 4397 smokers who participated in a two-armed RCT assessing computer-tailored smoking cessation advice in the United Kingdom, Kale et al [50] reported that a higher reading level was associated with successful quitting (OR 1.62, 95% CI 1.19-2.21). Reid et al [51] evaluated smokers in Canada, the United Kingdom, Australia, and the United States from the first five waves (2002-2006/2007) of the International Tobacco Control Four Country Survey (35,532 observations from 16,458 respondents). They reported that smokers with a high education level were more likely to be abstinent for 1 and 6 months (OR 1.20, 95% CI 1.00-1.44 and OR 1.30, 95% CI 1.05-1.62, respectively).

### Limitations

Limitations of this study were discussed previously [11] and include limited representation of individuals who use Android smartphones, are employed less than 20 hours per week, and are not seriously thinking of quitting smoking. Regarding the smartphone platform, Ubhi et al [52] reported differing behavior among users of a smoking cessation app (SmokeFree28 app) between Android and iOS users, with iOS users being more likely to have made a quit attempt in the last 12 months and set their quit date on the day of registration, and Android users being more likely to have used smoking cessation medication in their quit attempt. Baseline intention to quit (Stage of Change) as well as factors associated with socioeconomic status have been documented as predictors for quit attempts and success in quitting smoking [46,47,49-51]. Collectively, these findings highlight the need for additional data on the Pivot program in members of these groups with limited representation in this study. 

In addition, although multiple efforts were taken to minimize the impact of participant study payments, including keeping individual payments under US $50, incorporating a several-week delay between questionnaire completion and payment receipt, and not linking payment to use of program components or smoking outcomes, we cannot exclude some influence of study payment on participant behavior.

Owing to the sequential nature of Pivot, we elected to obtain outcome data as participants advanced through the self-paced program. The final follow-up data in our study is from a mean 7.2 (SD 1.2) months postenrollment, a time point that we believe was reasonable to consider with 6-month outcomes from other studies. This approach is different from the more traditional 30-, 90-, and 180-day assessments that are linked directly to enrollment date, and this difference is worth acknowledging as it limits direct comparison between studies.

The self-reported nature of smoking status is also a possible limitation. Biochemical verification of results was not sought for a few reasons. First, this study comprised general population smokers and was conducted entirely remotely with all data collection performed electronically via online questionnaires and through the Pivot app. Based on these study characteristics, biochemical verification was not pursued in accordance with previous recommendations [26,53]. Second, the breath sensor was employed as an educational and motivational tool in this study. We were concerned that implementing verification of smoking status with the breath sensor would instill a sense of policing that might detract from participant perception and experience of the sensor. Finally, while we acknowledge the possible limitation of over-reporting cessation with self-reporting smoking status, the literature suggests that the rate of occurrence is less than 10% in general population smokers. Specifically, Gorber et al [54] performed a systematic review of 54 studies to measure the concordance between self-reported smoking status and smoking status biochemically verified through measures of cotinine. The mean difference between self-reported and measured prevalence estimates was −4.8% for studies that measured cotinine in saliva; −6.2% for those measuring cotinine in serum, blood, or plasma; and −9.4% when cotinine was measured in urine.

Finally, although participants were provided education about NRT via the Pivot app and coaching in the study, they were not provided NRT. Given the well-established positive impact of NRT on smoking cessation [5,18-21], inclusion of NRT in Pivot would have likely further increased quit rates. The provision of NRT has since been added to the Pivot program; accordingly, future studies are warranted.

### Conclusions

This follow-up study provides the first longer-term outcomes of Pivot, an inclusive and innovative smoking cessation program that employs a smartphone app, biofeedback through a personal portable CO breath sensor, and in-app text-based coaching. At final follow up, approximately 7 months after enrollment, quit rates increased and continuous abstinence was favorable. Most participants made a quit attempt. The emergence of newly abstinent participants and persistent decreases in CPD 3 months after active program participation underscore the sustained learning and impact from Pivot. These results validate earlier findings, and suggest that Pivot is an effective and durable solution for smoking cessation.

## Abbreviations

CASC: CureApp Smoking Cessation
CO: carbon monoxide
CPD: cigarettes per day
FDA: Food and Drug Administration
IRB: Institutional Review Board
ITT: intention to treat
NRT: nicotine replacement therapy
OR: odds ratio
PPA: point prevalence abstinence
RCT: randomized controlled trial
RR: relative risk
USCPG: United States Clinical Practice Guideline
